# A Review on Oxidative Stress, Diabetic Complications, and the Roles of Honey Polyphenols

**DOI:** 10.1155/2020/8878172

**Published:** 2020-11-23

**Authors:** Visweswara Rao Pasupuleti, Chandra Sekhar Arigela, Siew Hua Gan, Sirajudeen Kuttulebbai Nainamohamed Salam, Kumara Thevan Krishnan, Nurhanan Abdul Rahman, Mohammad Saffree Jeffree

**Affiliations:** ^1^Faculty of Agro-Based Industry, Universiti Malaysia Kelantan, Campus Jeli, 17600 Jeli, Malaysia; ^2^Department of Biomedical Sciences and Therapeutics, Faculty of Medicine & Health Sciences, Universiti Malaysia Sabah, Malaysia; ^3^Department of Biochemistry, Faculty of Medicine and Health Sciences, Abdurrab University, Jl Riau Ujung No. 73, Pekanbaru 28292, Riau, Indonesia; ^4^School of Pharmacy, Monash University Malaysia, Jalan Lagoon Selatan, 47500 Bandar Sunway, Selangor, Malaysia; ^5^Department of Basic Medical Sciences, Kulliyyah of Medicine, International Islamic University Malaysia (IIUM), Bandar Indera Mahkota, 25200 Kuantan, Pahang, Malaysia; ^6^Department of Community and Family Medicine, Faculty of Medicine and Health Sciences, Universiti Malaysia Sabah, Kota Kinabalu, 88400 Sabah, Malaysia

## Abstract

Despite the availability of various antidiabetic drugs, diabetes mellitus (DM) remains one of the world's most prevalent chronic diseases and is a global burden. Hyperglycaemia, a characteristic of type 2 diabetes mellitus (T2DM), substantially leads to the generation of reactive oxygen species (ROS), triggering oxidative stress as well as numerous cellular and molecular modifications such as mitochondrial dysfunction affecting normal physiological functions in the body. In mitochondrial-mediated processes, oxidative pathways play an important role, although the responsible molecular mechanisms remain unclear. The impaired mitochondrial function is evidenced by insulin insensitivity in various cell types. In addition, the roles of master antioxidant pathway nuclear factor erythroid 2-related factor 2 (Nrf2)/Kelch-like ECH-associated protein 1 (Keap1)/antioxidant response elements (ARE) are being deciphered to explain various molecular pathways involved in diabetes. Dietary factors are known to influence diabetes, and many natural dietary factors have been studied to improve diabetes. Honey is primarily rich in carbohydrates and is also abundant in flavonoids and phenolic acids; thus, it is a promising therapeutic antioxidant for various disorders. Various research has indicated that honey has strong wound-healing properties and has antibacterial, anti-inflammatory, antifungal, and antiviral effects; thus, it is a promising antidiabetic agent. The potential antidiabetic mechanisms of honey were proposed based on its major constituents. This review focuses on the various prospects of using honey as an antidiabetic agent and the potential insights.

## 1. Introduction

Diabetes mellitus is characterized mainly by hyperglycaemia occurring due to defects in insulin secretion, insulin action, or both. According to the International Diabetes Federation (IDF), 463 million people globally have been diagnosed with diabetes in 2019 and the figure is expected to rise to 700 million by 2040 [1]. In fact, type 2 diabetes mellitus (T2DM) is one of the leading causes of illness and premature death in the world, with 4.2 million fatalities reported in 2019 [1]. The disease mainly affects both developed regions (“Occidental World”), as well as developing countries, contributed by unhealthy lifestyles, such as physical inactivity and high-fat and sugar consumption [2] .

In Asia, the numbers of diabetics in China (>113.9 million diabetics), India (>62 million), and Malaysia (3.5 million) render the region a critical “hot spot” for diabetes [3]. Some of the factors contributing to the accelerated trajectory of T2DM include (i) poor nutrition in utero and in early life and (ii) overnutrition in later lives (especially in populations undergoing rapid nutrition transitions, involving changed food habits and reduced physical activity). Interestingly, the prevalence of T2DM is slightly higher in men than in women [4].

Besides the environment, T2DM is a multifactorial disease involving genetic influence that has a “nature vs. nurture outcome.” The pathophysiological changes of diabetes are characterized by (1) *β*-cell dysfunction in the periphery (e.g., the liver, skeletal muscle, and adipose tissue) and (2) inflammation-dependent ROS generated locally by tissue or due to immune cells interacting with the insulin receptor (IR) as well as their downstream signalling pathways, resulting in a failure to respond adequately to insulin levels. All of these factors form a basis for insulin resistance and chronic inflammation which progressively hamper blood glucose control, leading to the development of micro- and macrovascular complications.

With respect to hyperglycaemia, it has been reported that at least eight distinct pathophysiological abnormalities contribute to impaired glucose homeostasis; this has been well established early in the natural history of T2DM [5]. In addition to the “ominous octet,” two additional pathophysiological abnormalities, namely, (1) activation of inflammatory pathways and (2) impaired insulin-mediated vasodilation, contribute to muscle insulin resistance. More recently, our understanding of the pathophysiology of T2DM has been further aided by the discovery of novel disease biomarkers where high blood concentrations of proinflammatory cytokines, such as C-reactive protein (CRP), interleukin-6 (IL-6), and tumour necrosis factor (TNF) are associated with an increased risk of T2DM [6] .

Oxidative stress is a condition that emerges from the imbalance between the formation of reactive oxygen species (ROS) and the ability of the antioxidant system to neutralize these compounds. Increased oxidative stress is the result of enhanced ROS production and/or decreased ROS scavenging ability resulting in tissue damage [7]. In fact, the pathology of a number of diseases including diabetic, vascular, and neural disorders are greatly influenced by oxidative stress.

Antioxidants are compounds that can help reduce oxidative stress. They can be (1) endogenous (reduced glutathione and antioxidant enzymes such as catalase, superoxide dismutase, glutathione peroxidase, and reductase) or (2) exogenous (antioxidant vitamins like vitamins A, C, and E). Both types of antioxidants can help prevent the formation of free radicals by scavenging or promoting their decomposition [8]. Additionally, they can transform the reactive metabolites into less reactive molecules [9]. As further insights into the molecular underpinnings of this disease and its destructive sequelae are gained, more opportunities in the development of novel, targeted approaches for diabetes treatment, prognosis and, ultimately, prevention exist.

Natural products have become a powerful tool to combat against oxidative stress since phytochemicals provide the main source for antioxidants. These antioxidants help in improving insulin secretion and hepatic glycogen storage and reduce oxidative stress [10]. To date, researchers have observed that honey is useful in preventing oxidative damage to various organs, including the brain and the heart [11, 12]. In particular, the health benefits of stingless bee honey (SLBH) have gained considerable attention in recent years [13], with the Nrf2/ARE antioxidant pathway emerging as one of the promising mechanisms. In this review, the roles of insulin resistance, oxidative stress, honey as an antidiabetic, and the Nrf2/ARE pathway involved in T2DM, as well as the current state of efforts aimed at exploiting natural products to increase the number of armamentariums against diabetes are reviewed.

## 2. The Role of Insulin Resistance in Diabetic Complications

### 2.1. Insulin

Insulin is a pancreatic *β*-cell anabolic peptide hormone, where its mature forms occur in two stages [14]. Pre–pro-insulin is processed first, resulting in the formation of proinsulin [15] from the signal fragment. Following this, the centre fragment (C-chain-35aa) is cut off, forming dipeptides via two chains of disulphides (A-21aa, B-30aa) [15]. Insulin is an important protein, playing several roles in regulating carbohydrate and lipid metabolisms, where the postprandial rise in blood glucose levels is the most significant stimulus for its production. By enhancing insulin output as well as its effects on the effector cells (myocytes, adipocytes, and hepatocytes), glucose transport into the cells is enhanced, thus decreasing the blood glucose. The event is accomplished by enhanced translocation of insulin-dependent glucose carriers (GLUT), with GLUT4 found in skeletal muscle, hepatocytes, and adipocytes [16].

When the glucose level in the small intestine exceeds 30 mM, glucose transport to the inside of the pancreatic *β* cells will be initiated in an insulin-independent manner via GLUT2. GLUT2 promotes transportation of glucose based on a concentration gradient. In the cell, glucose is converted into glucose-6-phosphate, which minimises equalization of glucose concentrations and prolonged transport of glucose into the cells. Further, glycolysis of glucose-6 phosphate leads to the formation of ATP, the level of which increases slowly due to a constant glucose supply. ATP inhibits the potassium channel because it blocks the outflow of potassium (K+ ions) from cells. Therefore, the level of K+ ions increases in the cell, which becomes more and more electropositive until the membrane charges are aligned, and the membrane is depolarized. The voltage-dependent calcium channel activates due to depolarisation, thus encouraging cell ion influx of calcium ions (Ca^2+^). Ca^2+^ activates the ryanodine channel in the membrane of insulin-accumulating vesicles, causing migration and the release of insulin molecules into the cell membrane [17].

### 2.2. Insulin Signalling Pathway

The released insulin is involved in many metabolic processes, including (1) deposition of glycogen in the liver and skeletal muscle, (2) inhibition of lipolysis, (3) enhancement of lipogenesis, (4) suppression of gluconeogenesis in the liver, and (5) enhanced glucose absorption via the insulin receptor signalling pathway [18]. Signal transmission from the blood into the cell is complicated and is a highly integrated process where hormone binding to the insulin regulator (IR) occurs, leading to the formation of a major large protein signal complex below the surface of the cell membrane [19].

IRs are heterotetrameric glycoproteins which are composed of two extra- (ш) and intracellular (*β*) subunits. They exist primarily on the cell layer of metabolically active tissues including bone, liver, and fat. Mostly, they occur on the surfaces of metabolic tissues such as liver, muscle, and fat. Insulin binding by extracellular subunits contributes to IR dimerization, thus allowing binding of ATP to *β*-subunits [20] which triggers the activation of the tyrosine kinase catalytic regions in the cytoplasm [21]. In the first phase, receptor autophosphorylation occurs, followed by phosphorylation of several substrate proteins where the most significant proteins appear to be the insulin receptor substrate (IRS). Subsequently, phosphorylation happens on tyrosine residues. Then, IRS-phosphorylated proteins can trigger two significant signalling mechanisms: (1) The first significant signalling mechanism leads from Ras to the mitogen-activated kinase (MAPK), thus contributing to the expression and/or regulation of genes important for cell development and differentiation. (2) The second significant signalling mechanism involves phosphatidylinositol 3 kinase (PI3K), which induces AKT/PKB receptor phosphorylation and is required for insulin's metabolic activity.

**Figure 1:**
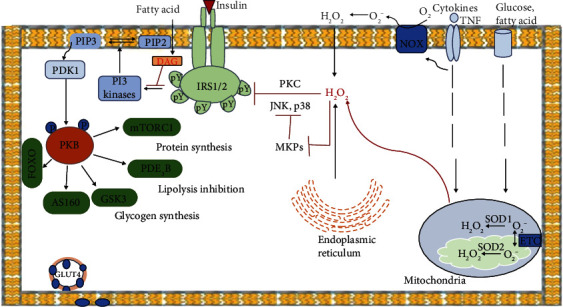
In the insulin signalling pathway, insulin attaches to insulin receptors triggering its dimerization and the intracellular autophosphorylation of their tyrosine residues, which constitute an attachment for IRS proteins. These molecules also undergo phosphorylation and form a complex with PI3K utilizing SH2 domains. PI3K phosphorylates PIP2, which results in the formation of PIP3 and activation of PDK1/2. AKT is then phosphorylated and activated by PDK1/2, subsequently eliciting phosphorylation of AS160. The latter is responsible for GLUT4 translocation to the cellular membrane as well as glucose inflow. Nutrient overload, inflammation, and endoplasmic reticulum (ER) stress contribute to the pathological generation of ROS in obesity and T2DM. The mitochondria are a primary source of oxygen (O_2_-), which is dismutated into hydrogen peroxide (H_2_O_2_) by superoxide dismutase 1 (SOD1) in the mitochondrial intermembrane space and SOD2 in the mitochondrial matrix. H_2_O_2_ diffuses across the membranes to promote the activation of Ser/Thr protein kinases such as PKC, MAPKs, JNK, and p38 (activation of which may be partly mediated by oxidation and inactivation of MAPK phosphatases (MKPs)), which phosphorylate IRS-1/2 to inhibit the association of IRS-1/2 with the IR, thus promoting IRS-1/2 degradation and suppressing the recruitment and activation of PI3K. Proinflammatory cytokines such as TNF promote mitochondrial ROS generation and activate nicotinamide adenine dinucleotide phosphate oxidase (NOX) enzymes on the plasma membrane to contribute to the development of insulin resistance.

Upon activation of the PI3K/AKT pathway, IRS proteins bind to PI3 kinase regulatory subunits via the SH2 domain. The PI3 kinase phosphorylates 4,5-biphosphate phosphatidylinositol (PIP2) and activates phosphatidyl inositol (3,4,5)-triphosphate (Pip3). This in turn contributes to the activation of the kinases of the PIP3-dependent kinases (PDK-1 and PDK-2) and ultimately subsequent activation of the AKT/PKB kinase and atypical protein kinase C (PKCs) [22]. Eventually, AKT then catalyses the phosphorylation of AS160 substrate proteins, which stimulates the translocation of GLUT glucose transporters from the cytoplasmic vesicles to the surface of the cell membrane, thus improving the transport of insulin-dependent glucose into the cell. GLUT4 occurs primarily inside the nonstimulated cells owing to two different actions: (1) slow exocytosis and (2) quick endocytosis. AS160 increases exocytosis of GLUT4 and inhibits its endocytosis into the adipocytes through its downstream target, Rab10, which results in the accumulation of GLUT4 in the plasma membrane [23]. In addition to activating insulin-dependent glucose uptake via GLUT4, AKT has many other intracellular targets and mediates various metabolic effects. For example, AKT triggers the phosphorylation of glycogen synthase kinase 3 (GSK3), allowing the liver and skeletal muscles to stimulate further glycogen synthesis as depicted in Figure 1 [24].

### 2.3. Insulin Resistance

Glucose is the primary energy source of our body, and is in fact, the only energy source for the brain. Nevertheless, homeostasis of glucose must be finely regulated with blood glucose levels maintained at specific levels (70-90 mg/dL). The key pathological features of T2DM are (1) insulin resistance, (2) a decrease in insulin response, and (3) a compensated increase in pancreatic *β*-cell insulin secretion to maintain euglycemia. Insulin resistance, if left unchecked, leads to the exhaustion of the *β*-cell and a reduced insulin secretion, leading to hyperglycaemia and acute diabetes.

Signalling for insulin deficiency is triggered by several factors, including IRS or downstream-located effector molecules as well as posttranslational changes in mutations and in insulin receptors. The most prevalent changes in insulin resistance include (1) a reduction in the number of insulin receptors and their catalytic activities; (2) enhanced Ser/Thr phosphorylation in the insulin receptors and IRS; (3) enhanced Tyr phosphatase activity, in particular PTP-1B involving receptors and IRS dephosphorylation; (4) lowered PI3K and AKT kinase activity; and (5) defects in functions and expression of GLUT4 [25]. These modifications cause a decrease in glucose uptake into the adipose and muscular tissues to stimulate metabolic changes and promote changes at a metabolic level.

Insulin resistance is distinguished by a series of intracellular changes such as (1) a decrease in receptor concentration and its kinase activity; (2) phosphorylation of IRS-1 and IRS-2; (3) PI3K activation; (4) activation of several other molecular factors that may be interrelated with the etiology of insulin resistance in liver, muscle, and fat; (5) modifications in mitochondrial respiration, inflammation, and endoplasmic reticulum (ER) stress; and (6) concomitant activation of Ser/Thr protein kinases, for example, the c-Jun N-terminal kinase (JNK), the I*κ*B kinase, (IKK), and the protein kinase C (PKC). Nevertheless, the key integrative mediators for the development of insulin resistance are thought to be the reactive oxygen species (ROS).

### 2.4. In Vitro Mechanism of Insulin Resistance Initiated by ROS

There are various mechanisms by which mitochondrial ROS may contribute to insulin resistance, with differing processes based on tissue type. ROS activates MAPKs such as JNK and p38. For example, ROS activates JNK by oxidizing and inactivating MAPK phosphatases (MKPs) (Figure 1), which dephosphorylate and inactivate JNK [26]. Additionally, ROS may oxidize thioredoxin (TRX), resulting in TRX dissociation and apoptotic signal-regulating kinase 1 (ASK1; kinase MAPK) complexes and a consequent JNK signalling activation [27]. Finally, the JNK inhibitor glutathione S-transferase Pi is oxidized by ROS and promotes JNK dissociation [28].

Although ROS activates IKK*β* via some mechanisms that remain unknown, in some cases, some mechanisms dependent on tyrosine phosphorylation may play a role. Specifically, Src protein tyrosine kinase ROS-mediated activation leads to activation of tyrosine phosphorylation and protein kinase D, which in turn phosphorylates and activates IKK*β* [29]. Regardless of the exact mechanisms by which JNK and IKK*β* are activated by ROS, several studies have revealed that the protein kinases contribute significantly to the development of insulin resistance in diabetes [30]. In the case of JNK deficiency, specific muscles prevent high-fat diet- (HFD-) induced muscle insulin resistance [31], while cell-permeable JNK inhibitory peptide administration improves glucose tolerance and causes increased insulin sensitivity as seen in obese db/db C57BLKS/J diabetes mice [32]. In the same way, the overexpression of hepatocyte-specific IKK*β* creates resistance to hepatic insulin [33], while IKK*β* hepatic impairment protects the mice against resistance to facilitate hepatic insulin as induced by HFD. [34]. Subsequently, JNK and IKK enter the insulin pathway during the IRS-1 phase [30]. IKK and JNK phosphorylate IRS-1 on serines 302, 307, and 612 to prevent the attachment of IRS-1 to the insulin receptor, thus promoting IRS-1 degradation and reducing the binding of phosphatidylinositol 3 kinase (PI3K) as well as the activation of important metabolic pathways, including activation of AKT. A study has shown that non-IRS-1-dependent tyrosine kinases receptors are also defective in AKT signalling when the cells become insulin resistant [35] indicating that while oxidative stress and increase of insulin resistance via IRS-1 Ser phosphorylation can happen in obese and diabetic states, it is possible that ROS and downstream IRS-1 serine phosphorylation will promote insulin resistance. Among the various proinflammatory cytokines, TNF-*α* is one of the most important proinflammatory cytokines, which promotes the development of insulin resistance and T2DM. In adipocytes and peripheral tissue, the high level of TNF-*α* from the macrophages of tissues [36] induces insulin resistance by altering insulin signalling via serine phosphorylation, again, leading to the development of T2DM [37].

Diacylglycerol- (DAG-) PKC signalling alterations in the muscle, liver, and adipose tissues are correlated with insulin resistance contributed by aging, high fructose, and fat feeding as well as obesity. Additionally, glucose-induced formation of DAG, which (1) improves phosphorylation, (2) decreases IR regulation, and (3) enhances glucose flux during hyperglycaemia occurring via the hexosamine biosynthesis pathway, can block glucose transport by activating PKC [38]. PKC has also been directly involved in lipolysis and metabolism of adipose tissue [39] where transfected hepatocytes that overexpress PKC tend to become resistant to insulin [40].

Various trials have shown a close relationship between ER stress, inflammatory response, and insulin resistance. ER stress is believed to contribute to the development of insulin resistance. Three stress-sensor kinases are activated in reaction to protein misfolding in the ER, namely, (1) protein kinase R-like endoplasmic reticulum kinase (PERK), (2) endoribonuclease inositol-requiring enzyme 1 (-1), and (3) activating transcription factor 6 (ATF-6), all of which detect protein misfolding and initiate a process recognized as unfolded protein response (UPR). These kinases also encourage processes that lead to the synthesis of inflammatory cytokines or activation of kinases that regulate IRS activity. PERK supports the release of NF-*κ*B from its inhibitor (I*κ*B), thus triggering its translocation to the nucleus, stimulating the production of defensive cytokines such as TNF-*α*, IL-1*β*, and IL-6 [41]. As a result, various Ser kinases including JNK [42, 43] and PKC-*θ* [44] are triggered, leading to IRS-1 phosphorylation on Ser, which results in insulin resistance. IRE-1 kinase phosphorylates and activates JNK, which in turn phosphorylates IRS-1 to Ser, an impact that decreases its ability to bind with other signalling proteins. With PI3K/AKT and MAP kinase pathways being impaired, insulin resistance occurs [45].

## 3. The Role of Oxidative Stress in Diabetic Complications

Although large amounts of cellular radical generation may be harmful, should there be a substantial rise in free radical generation or a reduction in free radical elimination from the cells, oxidative cellular stress occurs [46]. Therefore, it is essential to balance free radical generation with elimination. To date, there are sufficient experimental and clinical evidences that point to the increased formation of reactive oxygen species (ROS) in diabetes and that the development of diabetes is strongly linked to oxidative stress [47].

Oxidative stress causes elevated ROS and/or reactive species of oxygen (RNS) [47] which include (1) charged species like hydroxyl radical and superoxide and (2) uncharged species including peroxide of hydrogen and singlet oxygen [48]. The autooxidation of glucose; changes in redox balance; decreases in low-molecular-weight antioxidant substances like glutathione (GSH) and vitamin E; and impaired antioxidant defence operations, such as SOD and CAT, may be plausible causative factors of oxidative stress in diabetes [49]. Additionally, high-glucose-generated ROS is causally linked to elevated glucose and other metabolic abnormalities that are crucial to the development of diabetic complications. However, the precise mechanisms by which oxidative stress can lead to the progression of diabetic problems remain unknown [50].

In the last few decades, rising evidence has associated oxidative stress with a multitude of pathological conditions including cancer, cardiovascular disease, chronic inflammatory disease, postischemic organ injury, diabetes mellitus, and xenobiotic and rheumatoid arthritis [51]. The role of free radicals and oxidative stress in the pathogenesis and development of diabetic retinopathy, nephropathy, neuropathy, and rapid coronary artery disease has also been confirmed with convincing evidence [51]. In fact, several experiments have shown that increased extra- and intracellular glucose levels result in oxidative stress, both in experimental diabetes animals and in diabetic patients [51]. A cascade of ROS leaking from the mitochondria can be a source of oxidative stress which contributes to the (1) development of type 1 diabetes (T1DM) via apoptosis of pancreatic beta cells and (2) onset of T2DM through insulin resistance [52]. The fundamental processes in the development of diabetes are complicated, since hyperglycaemia may also be due to the cause-effect relationship of enhanced oxidative stress. Oxidative stress biomarkers are normally measured by lipid peroxidation indicators and protein oxidation, both in T1DM and T2DM [52].

Normally, oxidative stress in diabetes is contributed by various mechanisms, including excessive oxygen radical formation from the autooxidation of glucose [53], glycated protein formation, and antioxidant enzyme glycation which restrict the ability of antioxidants to detoxify the free radicals [54]. In contrast to these mechanisms, two other mechanisms have been proposed to be responsible for producing oxygen radicals in diabetes. First, Jain indicated that elevated glucose concentrations can boost cytochrome P450-like activity due to the production of excessive nicotinamide adenine dinucleotide phosphate oxidase (NADPH) generated by glucose metabolism [55]. Secondly, ketosis, a classic and specific example of T1DM, may also enhance the generation of free radicals in diabetic patients [56].

Many reports have attributed the common pathogenic element in endothelial and beta-cell dysfunction to oxidative stress [30]. For example, beta-cell dysfunction caused by prolonged exposure to high glucose raised free fatty acid (FFA) levels [57]. Beta cells are especially susceptible to ROS because they are low in enzymes such as CAT, glutathione peroxidase (GPx), and SOD; they are also low in free radical quenching (antioxidants). It is therefore not surprising that oxidative stress can damage mitochondria and significantly blunt insulin secretion [58]. It has been shown that oxidative stress caused by a limited exposure from beta-cell preparations to hydrogen peroxide (H_2_O_2_) can increase the output of protein cycline-dependent kinase inhibitor 1 (p21) and decrease insulin messenger ribonucleic acid (mRNA), cytosolic adenosine triphosphate (ATP), and calcium flux in the cytosol and mitochondria [59]. In fact, many experimental data have demonstrated that different kinds of vascular cells can generate ROS under hyperglycaemic situations [54].

**Figure 2:**
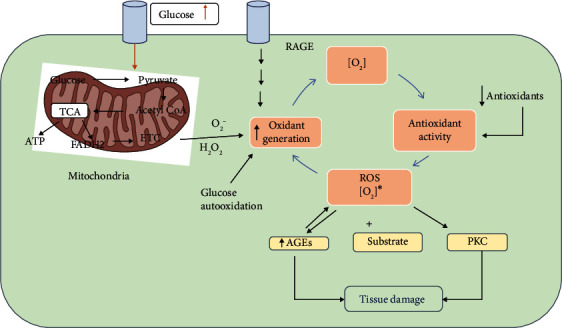
The relationship between rates of oxidant generation, antioxidant activity, oxidative stress, and oxidative damage in diabetes. [O_2_]^∗^ represents various forms of ROS. The overall rate of formation of oxidative products which lead to oxidative tissue damage is dependent on ambient levels of both [O_2_]^∗^ and substrate. Increased generation of [O_2_]^∗^ depends on several sources including glucose autoxidation, increased mitochondrial superoxide production, and increased endoplasmic reticulum superoxide production, as well as the result of the receptor for advanced glycosylation end product activation.[O_2_]^∗^ deactivation is reduced because antioxidant defences are compromised in diabetes. Note that oxidative stress also promotes other hyperglycaemia-induced mechanisms of tissue damage. Additionally, oxidative stress activates protein kinase C (PKC) and accelerates the formation of advanced glycosylation end products (AGEs).

ROS levels are tightly controlled by the antioxidant and nonenzyme antioxidant protective measures in normal and healthy cells. However, excessive ROS concentrations are contributed by hyperglycaemia in diabetes, leading to a significant complication of DM. In the case of diabetes or insulin resistance, a higher oxidative glucose metabolism itself increases the mitochondrial production of O_2_^•^, which is then converted to OH^•^ and H_2_O_2_ [60]. In addition to glucose, ROS generation by mitochondrial FFA is also augmented (Figure 2) [61]. Overall, the overexpression and activation of mitochondrial inner membrane uncoupling enzymes (UCPs) have been suggested to contribute to an increase in superoxide production under diabetic conditions [62].

NADPH oxidase is a significant source of ROS production in diabetes. NADPH oxidase is present in the plasma membrane of several renal cell types, including mesangial and proximal tubular cells, endothelial cells, fibroblast cells, and vascular smooth muscle cells. The production of NADPH oxidase-dependent ROS oxidation plays a major part in encouraging oxidative stress caused by hyperglycaemia. In fact, NADPH oxidase improves oxidative stress and ultimately leads to diabetic nephropathy in rats.

## 4. Antioxidant Defence System

In response to excess ROS production and oxidative stress during metabolism, organisms have developed multiple antioxidant mechanisms including antioxidants and enzymes. Superoxide dismutase (SOD), which is one of the most significant antioxidant enzymes, has three significant isoenzymes: (1) copper zinc- (CuZn-) SOD (SOD1), (2) manganese- (Mn-) SOD (SOD2), and (3) extracellular (SOD3). The main roles of SOD, CAT, and GPx are to detoxify O_2_- in H_2_O_2_ and water. Numerous other antioxidants are also available in cells that have significant roles in cell homeostasis, including glutathione and vitamins. Additionally, several unique antioxidant compounds such as vitamin E, lipoic acid, SOD mimetics, and N-acetylcysteine (NAC) have also been used to reduce complications of diabetes in mitochondria.

### 4.1. Mitochondria-Targeted Antioxidants

Mitochondria are undoubtedly one of the primary sources of ROS in human infections, and efforts have been made in the search of mitochondrial antioxidants. One of these studies is the synthesis of compounds linked with triphenyl phosphonium compounds by ubiquinol, ubiquinone, or vitamin E. The lipophilic cations are placed in the mitochondrial matrix where triphenyl methyl phosphonium cations [63] have also been used. The most commonly used compounds are MitoQ (ubiquinone) and mitovitamin E [49] which can accumulate a hundred times in the mitochondria [64] due to the membrane potential. Although the mechanism of action remains unclear, MitoQ has been used in humans as a pharmacological agent in Parkinson's disease and other diseases such as diabetes. In an animal study, MitoQ can be administered in drinking water to (1) protect against myocardial cell death, (2) enhance endothelial function, and (3) enhance mitochondrial respiration in rat models of ischemia reperfusion [65]. MitoQ decreases the amount of lipid peroxidation in rats by acting as a chain-breaking antioxidant [66]. It has sometimes been noted that MitoQ can act as a prooxidant agent [67] and confer some metabolic effects at elevated levels, possibly as a result of decoupling impact in mitochondrial complex I [68]. MitoQ may also act well on significant key enzymes including AMP kinase, pyruvate dehydrogenase, and phosphofructokinase.

### 4.2. Mitochondria-Targeted Antioxidant Peptides

There are other approaches to mitochondrial antioxidant treatment, although the mechanism of action which may include synthetic peptides with mitochondrial targeting and antioxidant effects [69] is clearly unknown. These agents may scavenge ROS and peroxynitrate (ONOO) and inhibit lipid peroxidation [69]. These agents, however, have a strong affinity to opioid receptors [70], thus limiting their clinical effectiveness such as in neuronal cell death [67], in pancreatic islet cells [71], and in rats fed a high-fat diet [69].

## 5. NRF2 Pathway

The expression of Nrf2 tends to increase in response to oxidative stress [72] since the transcription factor of Nrf2 has been recognized as a crucial molecular factor in orchestrating the adaptive cellular reactions under a broad range of cellular environments (either extracellular or intracellular) [73]. To date, both clinical and research communities are highly interested in understanding the upregulation of Nrf2 and/or its upstream antioxidant genes that are activated due to hyperglycaemia. The Nrf2 is a key regulator for cellular detoxification and redox status as well as for protection against oxidative stress [71]. Given that the molecular mechanisms for diabetic complications are not well understood, research can play a major role in explaining the molecular pathways and in developing strategies for macro- and microvascular diseases, including diabetes complications, prevention, therapy, and management.

### 5.1. Implications of Nrf2 Transcription Factor in Diabetic Complications

**Figure 3:**
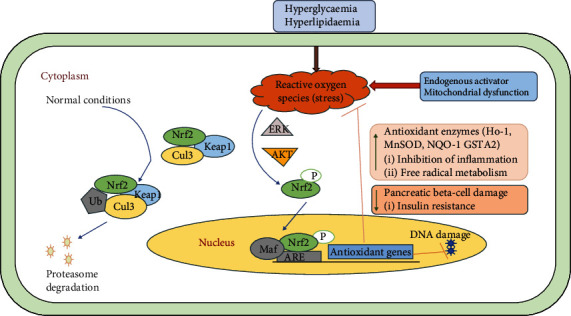
The Nrf2/Keap1/ARE pathway in T2DM. (a) Under diabetic conditions, the Nrf2 transcription factor is covalently bonded to cysteine residues on its native repressor Keap1 in the cytoplasm, which results in the (1) constitutive ubiquitination and degradation of Nrf2 in the proteasome and (2) inhibition of the antioxidant response. (b) During a stressful condition of electrophilic or oxidative stress, cysteine residues on Keap1 are modified, resulting in the stabilization and translocation of Nrf2 into the nucleus where it can bind to the promoter region of ARE and initiate the transcription of various cytoprotective enzymes which can promote cellular survival through a variety of mechanisms including the upregulation of antioxidant function, inflammatory inhibition, and the transport of toxic metabolites. These cellular adaptations have been shown to improve a wide array of tissue damage underlying the pathogenesis and progression of diabetes. There are three major mechanisms of Nrf2 induction by small molecule activators: (1) upstream kinases such as AKT and ERK, which phosphorylate Nrf2 at specific sites, favoring its release by Keap1 and nuclear translocation; (2) modification of Keap1 cysteine residues, which disrupts the Nrf2-Keap1 complex, favoring dissociation of Nrf2 and subsequent nuclear translocation; (3) inhibition of Nrf2 ubiquitination by Keap1 and degradation by the proteasome augments Nrf2 availability, thus favoring nuclear translocation of Nrf2. Ub: ubiquitination; P: phosphorylation.

Nrf2 gene, which is also known as NFE2L2, encodes for a transcription factor that controls for ARE genes in the promoter regions. These genes encode for proteins produced in response to environmental stress, metabolic enzymes, injury, and inflammation as well as detoxifying enzymes, including free radical production. Nrf2 in the cytoplasm is linked to Kelch-like ECH-associated protein 1 (Keap1) in unstressed circumstances, avoiding its translocation to the nucleus as illustrated in Figure 3 [72].

During stressed conditions (either electrophilic or oxidative stress), the Keap1/Nrf2 complex receives a signal involving phosphorylation and/or redox modification resulting in translocation of Nrf2 into the nucleus. Keap1, however, mediates the rapid ubiquitination and subsequent Nrf2 degradation by the proteasome 26S under basal circumstances. Cullin 3-based ubiquitin E3 (Cul-E3) ligase complex ubiquitinates Nrf2 while Keap1 acts as a substrate adapter which promotes the ubiquitination of Nrf2 by cullin 3. Consequently, Nrf2 has a short half-life (only 20 minutes) under normal circumstances. Oxidative stress is responsible for destroying critical cysteine residues in Keap1, disrupting the Keap1-Cul3 ubiquitination mechanism. However, if Nrf2 is not ubiquitinated, it builds up in the cytoplasm and is translocated to the nucleus (illustrated in Figure 3) [74, 75].

Nrf2 associates with the small protein called Maf present in its nucleus to create a heterodimer and initiate transcription in the upstream promoter region of several cytoprotective genes. The cytoprotective genes include the encoding antioxidant and phase-II detoxifying enzymes like superoxide dismutases (SODs), catalase (CAT), NAD(P)H dehydrogenase, *γ*-glutamyl cysteine synthase (*γ*-GCS), NQO-1, hemeoxygenase-1 (HO-1), glutathione peroxidase-1 (GPx-1), and glutathione S-transferase (GST). The response shown by the antioxidants to the signalling pathways NFE2L2 and NFE1-NFE2L2/ARE is considered a promising target against diabetic complications affecting the pulmonary, hepatic, digestive, neural, and cardiovascular systems as well as diabetic nephropathy [76, 77].

Animal studies have demonstrated that the Nrf2/Keap1/ARE system is a crucial defensive pathway to protect pancreatic *β*-cells both physiologically and pathologically. Nrf2 depletion can reduce cytoprotective antioxidant expression in *β*-cell transgenic mice and exacerbate oxidative *β* cell damage, where Nrf2 induction suppressed accumulation of ROS, ROS-induced DNA formations, and pancreative *β*-cell apoptosis in the islets [78]. In Nrf2/Keap1/ARE, pancreatic *β*-cell protection does not only include free radical scavenging activity but also an inflammatory reduction via the NF-*κ*B pathway [79]. A new study in murine models illustrates that increased signalling of Nrf2 can also improve insulin resistance via suppression of hypothalamic oxidative stress, a phenomenon that can affect systemic metabolic regulation [80]. Murine studies have also confirmed that Nrf2 induction can suppress weight gain and increase the consumption of skeletal muscle oxygen, mitochondrial redox homeostasis, and ATP production as well as increase the intake of cellular glucose in type II diabetes [81].

## 6. Honey Polyphenols as Antidiabetic Agents

Scientists look into alternative medicines for diabetic treatment because of the adverse effects conferred by conventional allopathic treatments as well as the economic burden of the conventional diabetic medications. Natural products have always been used in the treatment of diabetes, many of them being polyphenols which are bioactive [82–86]. Dietary flavonoids have been considered a related risk-reduction factor for T2D [87]. For many years, people have believed that diabetic patients cannot consume honey due to its high sugar content. However, a series of questions have emerged: “Can honey replace sugar in diabetic diet?” “Is the sugar in honey key in prevention and treatment of diabetes mellitus?” Interestingly, several researchers worldwide have focused on the characterization of honey from different sources and determining its biological properties. This study provides stronger evidence to support the function of fructose in mediating the hypoglycemic effect of honey. Fructose and glucose are the major monosaccharides with the same molecular formula but with a different structural formula present in honey [88]. Several studies reported that fructose reduces glucose levels or hyperglycaemia in diabetes patients and diabetic rat models [42, 89–91]. Evidence showed that gastric emptying is prolonged by the intake of fructose [92], which may slow down the rate of intestinal absorption [93]. In addition to delaying absorption, fructose intake reduces the intake of food [11], which is often due to delayed gastric emptying [94]. Fructose does not increase plasma glucose and does not require insulin secretion to its metabolism. Glucokinase, a vital enzyme for the intracellular metabolism of glucose, is known to be activated by dietary fructose [95]. Glucokinase further catalyses glucose converted to glucose-6 phosphate, which decreases blood glucose [17], and fructose stimulates secretion of insulin from an isolated pancreas [96]. Curry et al. observed no insulin reaction to fructose in rat pancreas preparation when very low glucose is present in the medium; however, when higher glucose concentration is present, insulin reaction to fructose was observed. Various preclinical and clinical studies have highlighted the efficacy and use of honey in diabetic patients and in animal models [97–100]. Nevertheless, although the hypoglycemic effect of honey remains unclear, numerous experiments have indicated that it is so [100, 101].

Although honey holds several types of polyphenols, only a few, such as kaempferol, catechin, quercetin, luteolin, rutin, and apigenin, have been shown to decrease blood glucose levels through various mechanisms [102]. Some of the mechanisms include (1) inhibition of enzymes such as *α*-glucoside and *α*-amylase [81]; (2) improved pancreatic *β*-cell protection by (i) reducing oxidative stress, (ii) increased secretion, and (iii) increased insulin activity [85]; (3) higher absorption of glucose and production of insulin receptor and GLUT4; (4) inhibition of gluconeogenic enzymes; and (5) inhibition of the enzyme aldose reductase. Plasma glucose levels have significantly been reduced due to honey administration and as a result of enhanced insulin secretion. In an experiment on normal rats which received honey supplementation (10%), the glycated haemoglobin was considerably reduced [103], where *α*-glucosidase and *α*-amylase were inhibited, the crucial enzymes involved in ameliorating blood glucose level elevation as a result of carbohydrate breakdown. The effect is purported to be the effect of honey polyphenols and other polyphenols as proposed by several investigations [83, 104]. Thus, the inhibition of both enzymes is considered an efficient method to decrease blood sugar concentrations in diabetic patients [104].

A few experiments using animal models have reported some reduction in blood glucose due to the presence of fructose, which may contribute to the decreased food intake, reduced rate of intestinal absorption, and a prolonged gastric emptying time. Fructose triggers glucokinase's activity in the hepatocytes, involved in both the absorption and storage of glucose by the liver as glycogen. Glucose, unlike fructose, enhances the absorption of fructose and improves hepatic function by enhancing fructose delivery to the liver.

The pancreas is one of the most important organs involved in diabetic management, due to the fact that insulin and glucagon are produced by the pancreatic cells. The hypoglycaemic effect of honey may be attributed to its antioxidant molecules that help defend against oxidative stress and damage. Insulin response and glucose homeostasis in normal rats are enhanced by fructose intake or by a combination of sucrose molecules in comparison with glucose intake in rats. Notably, it has been demonstrated that honey administered in sufficient doses, can confer hypoglycaemic effects in animal models of types 1 and 2 diabetes as, respectively, induced by alloxan and streptozotocin [100, 105, 106].

In another study, honey and fructose were fed to type 1 diabetic (alloxan-induced) and healthy rats. The former showed a significant reduction in glucose levels, when compared to the latter in control rats [107, 108]. It was also shown that honey effectively brought about glucose homeostasis when given in the diet for diabetics (or hypertriglyceridemia) and in healthy patients in contrast to a diet that is rich in sucrose and dextrose. In diabetic patients, plasma glucose levels were substantially decreased with honey administration when compared to dextrose consumption. Interestingly, it was observed that in normal subjects, C-reactive protein, blood lipids, and homocysteine were reduced after regular consumption of honey when compared to sucrose consumption [108, 109].

Honey can noticeably reduce fructosamine concentration which is a glucose-modified protein; this is an added metabolic advantage for diabetes mellitus and is not generally yielded with other hypoglycaemic agents. Fructosamine can undergo oxidative cleavage resulting in the formation of ROS and has been shown to be substantially increased in rats with diabetes mellitus and/or their complications [110], implicating that fructosamine is an important molecular player in diabetes. Antioxidants in honey scavenge the free radicals crucial in various inflammatory cascades and suppress the generation of reactive oxygen species (ROS) catalysed by metal ions such as iron and copper. These metal ions can be reached with flavonoids and other polyphenolic compounds found in honey [111]. Thus, honey possesses an antioxidant activity mediated by flavonoids and polyphenols. Additionally, honey can mediate insulin secretion due to the presence of minerals including zinc, manganese, copper, selenium, chromium, calcium, and potassium, which implies the antidiabetic activity of honey [112]. A clinical research [113] has demonstrated that early modifications of special trace elements can lead to impaired insulin and glucose metabolisms, while on the other hand, diabetes mellitus is said to be linked to trace element disturbance [113].

Deficiencies in some elements such as zinc (Zn), chromium, and manganese can enhance an individual's risk of glycemia and diabetic complications. For example, Zn is directly engaged with insulin synthesis, storage, secretion, and conformational integrity. Zn assembles into crystalline insulin as a dimeric structure for storage and secretion. In people with type-2 diabetes mellitus, a lower zinc concentration may influence the capacity of pancreatic cells to synthesize and secrete insulin [114].

T2DM can be induced by streptozotocin (STZ) by damaging *β*-cells of islets of Langerhans in the pancreas. These cells produce insulin to enhance glucose cellular uptake. There was a significant decrease in blood glucose concentrations three days following administration of Mad honey produced from the flower of rhododendrons in STZ-induced diabetic rats and nondiabetic rats [115].

Honey's antidiabetic effect is also explained by its antioxidant capacity and modulation of adiponectin concentrations. Adipose tissue secretes adiponectin hormone, which regulates both glucose and lipid metabolisms and is shown to be significantly reduced in diabetic patients [116]. Importantly, oxidative stress-mediated lipid peroxidation was directly implicated in the development of diabetic complications [117]. High concentrations of adiponectin decrease systemic inflammation and increase insulin sensitivity [118].

In another type of honey, Gelam honey extracts were used to protect the pancreatic hamster cells from hyperglycaemic conditions. Gelam honey significantly reduced production of ROS, glucose-induced lipid peroxidation, increased insulin content, and cell viability under hyperglycaemic conditions [119]. Additionally, Jujube honey has been shown to modulate the key enzymes involved in glucose metabolism, namely, glucokinase and glucose 6-phosphatase in rats. It was observed that Jujube honey reduced malondialdehyde (MDA) levels but significantly increased total antioxidant capacity in diabetic rats (*p* < 0.05). Interestingly, heat shock protein (HSP70) and glucose expression of 6-phosphatase were reduced as well, while glucokinase expression was increased in the treatment group [120].

In 2015, Hemmati et al. have demonstrated that honey orally administered (1.0 and 2.0 g/kg/day) to STZ-induced diabetic rats for 21 days has resulted in a substantial rise in adiponectin concentrations (by 4.5 ± 0.2 and 4.2 ± 0.3 mg/L, respectively) with a marked reduction in malondialdehyde (MDA) concentrations as compared to control rats. The increase is linked to a substantial enhancement in the level of fasting blood sugar (FBS) and lipid profiles in honey-supplemented diabetic rats [121].

Multiple evidences have suggested that the antidiabetic and hypoglycaemic abilities of honey is dependent on its antioxidant capacity; indeed, the pathogenesis of diabetes mellitus, especially that of type 2, appears to be closely linked to oxidation stress and ROS in different organs and tissues [122]. A rise in glucose absorption by the adipose tissues and muscles increases ROS production, leading to oxidative stress, a process that affects glycogen synthesis and glucose uptake. Oxidative stress may also lead to insulin resistance due to insulin signalling impairment, which can be restored through honey treatment [123].

In pancreatic *β*-cells, oxidative stress affects cell functionality, resulting in inadequate insulin secretion and a high rate of *β*-cell apoptosis. The scavenger activity of honey is normally confirmed to enhance pancreatic oxidative stress [124]. Lipid metabolism is impaired in diabetes mellitus, which shows a high concentration of low-density lipoproteins (LDLs), which are oxidized and glycated in oxidized LDLs, thus leading to endothelial damage. In this case too, honey antioxidants help to inhibit lipid oxidative degradation in patients with type 2 diabetes mellitus [125].

The antioxidant impact of Tualang honey (TH) in the pancreas of diabetic rats induced by STZ has been explored. Following supplementation of TH (1.0 g/kg/day) for 28 days, there was substantial downregulation of SOD and MDA, together with increased pancreatic CAT activity (*p* < 0.05) in diabetic rats as compared to control rats. It is plausible that the antioxidant effects of TH protected the pancreatic cells from oxidative stress which led to an enhancement of FBS in diabetic rats when compared with control rats (median (IQR): 8.8 (5.8) and 17.9 (2.6) mmol/L, respectively) [126]. Similar hypoglycaemic impact is also exerted by other types of honey, such as honey from Nigeria. In fact, alloxan-induced diabetic rats supplemented with honey for 21 days at two doses (1.0 and 2.0 g/kg/day) had considerably (*p* < 0.05) decreased FBS as compared to control [127].

In another study, honey collected from Ilam forest, Ilam province, Iran, has been shown to enhance glycemic control. Consumption of natural honey (1.0 g/kg) along with metformin (100 mg/kg) once daily for four weeks tends to be more effective than administering metformin alone (100 mg/kg). Ilam honey was shown to be rich in antioxidants and is more effective than Tualang honey in treating lipid abnormalities [128].

**Figure 4:**
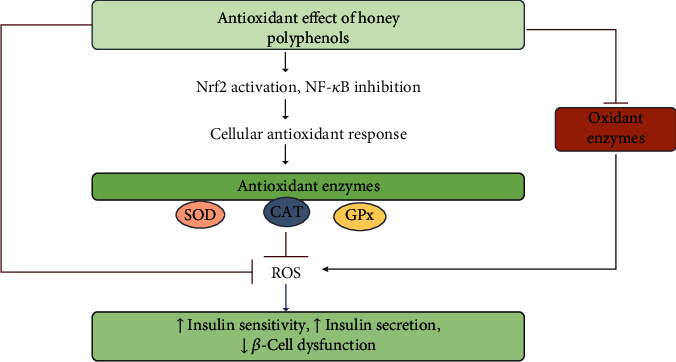
Potential mechanisms contributing to the beneficial effects of polyphenols by protecting cells against oxidative stress. Abbreviations: CAT—catalase; GPx—glutathione peroxidase; NF-*κ*B—nuclear factor kappa light-chain enhancer of activated B-cells; Nrf-2—nuclear factor erythroid 2; ROS—reactive oxygen species; SOD—superoxide dismutase.

It has been shown that polyphenols present in honey have protective activity on pancreatic *β*-cells, thus alleviating diabetes. In streptozotocin-induced diabetes in rats, the regeneration of pancreatic *β*-cells improved by administering quercetin at a dose of 10-15 mg/kg for ten days [129–131] and increased glucose uptake in insulin-resistant tissues [132]. Apigenin and rutin were shown to have guarded pancreatic *β*-cells by elevating the levels of antioxidants, antioxidant enzymes, and insulin in rat models of streptozotocin-induced diabetes [133]. In another research, quercetin protected and retained the structure and integrity of the pancreatic *β*-cells while improving insulin secretion in streptozotocin-induced diabetes in rats [130]. Kaempferol, a flavanol found in honey, was reported to have prevented pancreatic *β* cell dysfunction in middle-aged obese mice with diabetes, and enhanced peripheral insulin sensitivity [131]. Clinical studies indicate that both honey and other sources of polyphenols decrease oxidative stress in diabetic animals (Figure 4). Quercetin, found in honey, decreased oxidative stress in streptozotocin-induced diabetes in rats by lowering blood glucose concentrations and lipid peroxidation, and raising levels of ascorbic acid, vitamin E, and tissue antioxidant activity [134]. Luteolin is a flavone, reported to be present in honey, which has been shown to reduce oxidative stress in type 1 diabetic cardiomyopathy to ameliorate heart failure [135].

In DM, glucose usage is reduced in both insulin and non-insulin-susceptible tissues. Dietary polyphenols found in honey are reported to boost peripheral absorption of glucose into these tissues. Two other plant phenolic compounds also discovered in honey are chlorogenic and ferulic acids that can increase glucose uptake and be more efficient as two hypoglycaemic medicines called metformin and 2,4-thiozolodinedione (THZ) [136]. Chlorogenic acid stimulates glucose uptake through the expression of the PI3K-independent pathways of the GLUT4 and peroxisome proliferator-activated gamma receptors (PPAR-*γ*). Ferulic acid, however, promotes glucose uptake by expressing GLUT4 and PI3K transcripts via PI3K process-dependent pathways [136].

Improving the activity of insulin secretion is the most significant goal in the treatment of DM. It was demonstrated that daily administration of rutin 25-100 mg/kg for 45 days could enhance insulin secretion and reduce fasting blood glucose concentrations in streptozotocin-induced diabetes in rats [137]. Quercetin protected pancreas *β*-cells against hydrogen peroxide (H_2_O_2_) and the activation of ERK-1/2 (extracellular signal-regulated kinases) pathway triggered by glucose and glibenclamide in insulin-secreting cell lines (INS-1) [138]. Luteolin, a flavonoid, was shown to reduce hyperglycaemia by raising insulin secretion in a diabetic animal model [139]. The process of activation of peroxisome proliferator-activated receptor gamma (PPAR-*γ*) [140] also prevented insulin resistance in T2DM patients by the formation of adipokines, which implies that honey may be helpful in modulating adiponectin mediated by PPAR-*γ* [116], thus alleviating insulin resistance.

A nonrandomized open clinical trial single-arm phase I cohort prospective study on the effects of honey reported that honey consumption resulted in hyperglycaemia in type 2 diabetic patients. Interestingly, regular and long-term honey consumption was shown to decrease the body weight and blood pressure in the same cohort [141] of subjects. Another randomized controlled trial was conducted in type 2 diabetics to investigate the effect of daily ingestion of kanuka honey blended with “cinnamon,” “chromium,” and “magnesium” on glucose metabolism and lipid variables [142]. However, the blended honey supplementation did not produce any statistically significant change in HbA1c or fasting insulin in the subjects, although the supplementation has alleviated abnormal lipid variables, improved high-density lipoprotein, and led to a reduction in body weight. The two studies have shown contrasting findings on the antidiabetic activity of honey, although they also showed lipid profile improvement and body weight reduction in the subjects. However, further clinical studies have to be conducted to gain insights into the antidiabetic activity of honey with more stringent criteria to select honey and subjects for inclusion in the studies.

Based on the data from the experimental studies and reports, it is clear that honey is useful in the treatment of diabetes mellitus patients. Although some studies have suggested contrasting views on the usage of honey, most scientists agree that honey is useful in improving hyperglycaemia and diabetic problems on various organs and may reduce complications.

## 7. Conclusion and Future Directives

The burden in the community is increasing with chronic illnesses including diabetes mellitus, hypertension, atherosclerosis, and cancer. The mortality resulting from these diseases has also increased. The evidence that oxidative stress plays a substantial role in pathogenesis or disorders suggests that antioxidants can be beneficial. However, a variety of antidiabetic drugs may also contribute to cardiovascular complications. Natural substances have been used for a long time to cure different types of diseases, including T2DM. Honey is a natural product, and it has several biochemical and biological roles in both humans and animals. Honey is a major source of polyphenols, and the level of each polyphenol varies considerably. The polyphenols inhibit the development of different diseases through several specific mechanisms, for example, regulating the expression of a certain gene or encouraging/blocking certain steps on a metabolic pathway. Various types of honey have been investigated for their antimicrobial, antidiabetic, anti-hyper-cholesterol, anti-inflammatory, antioxidant, and wound-healing effects. Moreover, their credibility is limited owing to a relatively low number of clinical trials. Further investigation is required to determine specific mechanisms. It is highly imperative that several clinical and experimental trials are carefully designed and planned since they are often required to verify the efficacy of honey either alone or as an adjuvant treatment.
